# A latent scale model to minimize subjectivity in the analysis of visual rating data for the National Turfgrass Evaluation Program

**DOI:** 10.3389/fpls.2023.1135918

**Published:** 2023-07-06

**Authors:** Yuanshuo Qu, Len Kne, Steve Graham, Eric Watkins, Kevin Morris

**Affiliations:** ^1^ National Turfgrass Evaluation Program, Beltsville, MD, United States; ^2^ U-Spatial, University of Minnesota, Minneapolis, MN, United States; ^3^ U-Spatial, University of Minnesota, Duluth, MN, United States; ^4^ Department of Horticultural Science, University of Minnesota, St. Paul, MN, United States

**Keywords:** NTEP, visual ratings, cultivar evaluation, subjectivity minimization, Bayesian model

## Abstract

**Introduction:**

Traditional evaluation procedure in National Turfgrass Evaluation Program (NTEP) relies on visually assessing replicated turf plots at multiple testing locations. This process yields ordinal data; however, statistical models that falsely assume these to be interval or ratio data have almost exclusively been applied in the subsequent analysis. This practice raises concerns about procedural subjectivity, preventing objective comparisons of cultivars across different test locations. It may also lead to serious errors, such as increased false alarms, failures to detect effects, and even inversions of differences among groups.

**Methods:**

We reviewed this problem, identified sources of subjectivity, and presented a model-based approach to minimize subjectivity, allowing objective comparisons of cultivars across different locations and better monitoring of the evaluation procedure. We demonstrate how to fit the described model in a Bayesian framework with Stan, using datasets on overall turf quality ratings from the 2017 NTEP Kentucky bluegrass trials at seven testing locations.

**Results:**

Compared with the existing method, ours allows the estimation of additional parameters, i.e., category thresholds, rating severity, and within-field spatial variations, and provides better separation of cultivar means and more realistic standard deviations.

**Discussion:**

To implement the proposed model, additional information on rater identification, trial layout, rating date is needed. Given the model assumptions, we recommend small trials to reduce rater fatigue. For large trials, ratings can be conducted for each replication on multiple occasions instead of all at once. To minimize subjectivity, multiple raters are required. We also proposed new ideas on temporal analysis, incorporating existing knowledge of turfgrass.

## 1 Introduction

The National Turfgrass Evaluation Program (NTEP) is an internationally renowned turfgrass research program. Starting from 1981, NTEP has coordinated trials and collected data on a variety of turfgrass species at locations across the United States and Canada (Xie et al., 2022). At each testing location, replicated turf plots of different cultivars are established, maintained, and visually evaluated by trained raters periodically on various traits of interest. Experienced raters usually mentor new raters following rating guidelines set by NTEP. Evaluated traits have traditionally included overall quality, color, density, resistance to diseases and insects, tolerance to biotic or abiotic stresses, and more recently expanded to drought and traffic tolerance. Over the years, NTEP has created a unique data repository, providing rich information for characterizing and selecting turfgrass cultivars for various applications.

NTEP adopted a 1-9 integer scale to assess traits of selected turfgrass cultivars (hereinafter referred to as the NTEP scale). It was originally used by turfgrass researchers in the 1980s in the northeastern region of the United States (personal communication with Dr. Bill Meyer of Rutgers University), which resembles the 9-point hedonic scale. Developed by David R. Peryam and his colleagues (Peryam and Girardot, 1952; Peryam and Pilgrim, 1957), the 9-point hedonic scale was originally used to measure the food, i.e., the stimuli, preferences of soldiers, i.e., the subjects, in the U.S. Armed Forces in the 1950s. Since then, it has become the most widely used scale for testing consumer preferences and acceptability of foods and beverages (Lim et al., 2009). The original 9-point hedonic scale is a balanced bipolar scale centered around a neutral position with four positive and four negative categories on each side. The categories are labeled with phrases ranging from “Dislike Extremely” to “Like Extremely” (**Table 1**), representing a continuum from dislikes to likes.

**Table 1:** Replication of the questionnaire designed for studying soldiers**’** preferences in the field.

	FOOD ITEM	LIKE				INDIFFERENT	DISLIKE			
Not Tried	Cream Gravy	Like Extremely	Like Very Much	Like Moderately	Like Slightly	Neither Like Nor Dislike	Dislike Slightly	Dislike Moderately	Dislike Very Much	Dislike Extremely
Not Tried	Bread Putting	Like Extremely	Like Very Much	Like Moderately	Like Slightly	Neither Like Nor Dislike	Dislike Slightly	Dislike Moderately	Dislike Very Much	Dislike Extremely
Not Tried	Cheese	Like Extremely	Like Very Much	Like Moderately	Like Slightly	Neither Like Nor Dislike	Dislike Slightly	Dislike Moderately	Dislike Very Much	Dislike Extremely
Not Tried	French Fried Onions	Like Extremely	Like Very Much	Like Moderately	Like Slightly	Neither Like Nor Dislike	Dislike Slightly	Dislike Moderately	Dislike Very Much	Dislike Extremely
Not Tried	Lettuce Wedges	Like Extremely	Like Very Much	Like Moderately	Like Slightly	Neither Like Nor Dislike	Dislike Slightly	Dislike Moderately	Dislike Very Much	Dislike Extremely

Response to the 9-point hedonic scale is an ordinal variable as its categories have a natural order (Seddon et al., 2001). In subsequent analysis, the categories are generally assigned with numerical values from 1 to 9, respectively, such that parametric statistical models can be utilized. For the NTEP scale, a trained rater walks through all plots in serpentine order in each rating event, assigning an integer from 1 to 9 directly for a particular trait of interest where 1 is typically the poorest/lowest and 9 is the best/highest. Similar to analyzing responses to a 9-point hedonic scale, the analysis of NTEP rating data treats the ordinal variables as numerical values, which may lead to serious errors, such as increased false alarms, i.e., detecting non-existing effects, failures to detect effects, and even inversions of differences among groups (Bürkner and Vuorre, 2019). There is abundant literature, e.g., Lim et al. (2009), Liddell and Kruschke (2018), on the reasons for these problems. Some important ones are summarized here.

1. The categories in the 9-point hedonic scale are not equidistant, which was first discovered by the Psychometric Laboratory at the University of Chicago (Jones and Thurstone, 1955; Jones et al., 1955), and confirmed in later studies (Moskowitz, 1971; Moskowitz and Sidel, 1971; Moskowitz, 1977; Moskowitz, 1980).
2. The 9-point hedonic scale lacks an absolute zero point. While there is a neutral position (i.e., the INDIFFERENT category or the "5"), it varies from subject to subject, even across different measurements by the same subject.
3. The general tendency of subjects to avoid using the extreme categories (Hollingworth, 1910; Stevens and Galanter, 1957; Parducci and Wedell, 1986) makes the scale vulnerable to ceiling and flooring effects. This truncates the 9-point scale, limits the scale’s ability to identify extreme stimuli, and skews the response data.

As a derivation of the original 9-point hedonic scale, the NTEP scale also yields ordinal data. Such data only provide rudimentary information on the hedonic magnitude and cannot directly be used to compare hedonic perceptions across different raters. In the current evaluation process, a turf plot’s rating for a specific trait, e.g., turf quality, depends on the rater’s severity in the rating event. Given the same plot, it will likely score higher when the rater is lenient or lower when severe, giving rise to subjectivity. In other words, for a specific rater’s turf quality ratings, we know a “3” plot has better turf quality than a “2” plot. But we cannot conclude a “3” plot rated by A is better than a “3” plot rated by B in turf quality without adjusting for rater severity. Considering the temporal nature of the evaluation process, even for the same rater on the same trait, consistency is not guaranteed at different times of the year. Another source of subjectivity relates to the scale categories, which are not equal distances or of the same levels. To meaningfully aggregate data collected from different rating events across different testing sites, both sources of subjectivity need to be addressed. However, current methods, e.g., the additive main effect and multiplicative interaction (AMMI) method, analysis of variance (ANOVA) (Ebdon and Gauch Jr., 2002a; Ebdon and Gauch Jr., 2002b), and linear mixed model (LMM), are not adequate and suffer the same errors when they were applied to ordinal data directly. Inspired by Rasch Rating Scale Model (Andrich, 1978), we propose a latent scale model to minimize subjectivity, hereinafter referred to as NTEP RSM (NTEP Rating Scale Model), allowing more objective comparisons of cultivars across different raters and research groups. We also demonstrate how to fit the described model in a Bayesian framework, using datasets on overall turf quality ratings in the 2017 NTEP Kentucky bluegrass trials. The model is programmed in Stan (Lee et al., 2017) *via* Python. Stan is a probabilistic programming language for statistical modeling, inference, and computation. Although demonstrations are done for overall turf quality rating, this approach works for other traits of interest evaluated using the 1-9 NTEP rating scale.

## 2 Model specifications

### 2.1 NTEP RSM

We started by constructing a latent scale based on the probability distribution of raw ordinal data. The model predicts the decision between two adjacent categories using a threshold parameter on the latent scale. The 1-9 scale is re-indexed in the following sections as 0-8 categories for conciseness in mathematical notations. At a given test location, let *Y_ni_* denote the rating assigned to plot *n* in rating event *i*, the logarithmic ratio of the probability of plot *n* assigned to category *s* to that of plot *n* assigned to *s*–1 can be expressed by the following equation,


(1) $$ln[\frac{Pr(Y_{ni}=s)}{Pr(Y_{ni}=s-1)}]=\theta_{n}-\beta_{i}-\tau_{s}$$

where

*i*=1,2,…,*I* is the index for each rating event during the trial;
*n*=1,2,…,*N* is the index for each plot;
*s*=1,2,…,*M* is the index for category thresholds;
*M*(*M ≤* 8) is both the maximum rating score after reindexing and the number of thresholds;
*θ_n_* is the perceived turf quality of plot *n* in a specific rating event;
*β_i_* measures rating severity in rating event *i*;
*τ_s_* is the threshold at which at *Pr*(*Y*=*s*–1) = *Pr*(*Y*=*s*).

**Figure 1:**
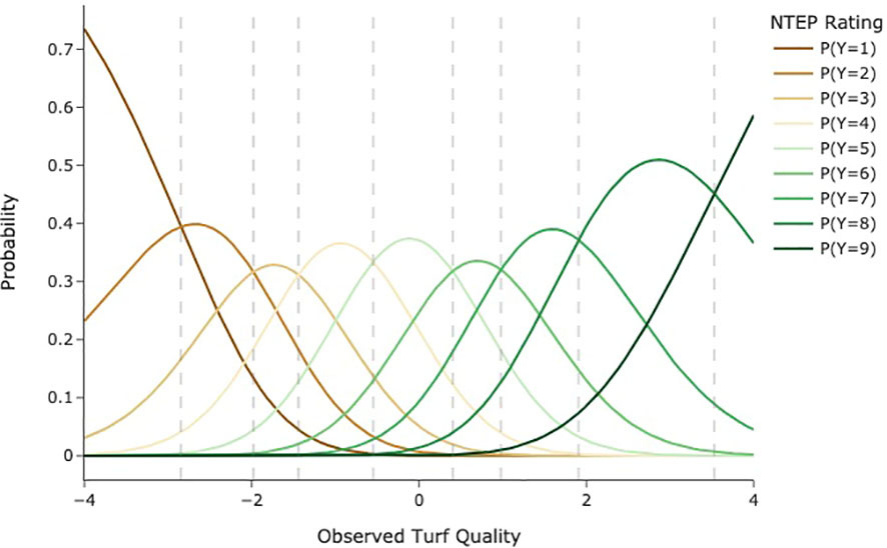
Hypothetical category probability curves for nine ordered categories as used in NTEP rating scale.


Constraints were placed on *β_I_* and *τ_S_* to add a meaningful zero to the scale. Both parameters were constrained to be the negative sum of the other parameters, respectively. We further assume ***θ***, ***β***, and *mbolτ* are normally distributed. For an unbiased rater in a rating event (*β=0*), the probability density curves for each category are illustrated in **Figure 1**. The vertical dash lines indicate category thresholds located at the points where the probability of a cultivar being assigned to two adjacent categories is equal. Note that these thresholds are not necessarily equidistant. In **Figure 1**, if a cultivar is located in a category (i.e., between two adjacent thresholds), then the response in that category has the greatest probability. The x-axis represents the constructed latent scale. It is continuous and equidistant, with a zero indicating the average level of overall turf quality. While the average level in individual rating events might vary (*β*≠0), we assume the average levels for each research group at different test locations are the same, allowing scale matching across different testing locations. Once subjectivity effects, i.e., ***β*** and ***τ***, were estimated and removed, ***θ*** can be further analyzed. In this study, we partitioned ***θ*** into cultivar and plot location effects, that is,

(2) θ=η+LOC

where ***η*** is the cultivar effect, reflecting the intrinsic quality of a cultivar, and LOC is the plot location effect due to spatial heterogeneity of the field. We further assume cultivar effects follow normal distributions with a mean of 0 and a variance of *σ* ^2^. The plot location effect was modeled as a Gaussian process with a zero mean and covariance function *K*,


(3) $$LOC\left(\cdot \right)∼N\left(0,K\left(\cdot \right)\right)$$

The covariance function *K*(·) implemented here is an exponential quadratic function. For two plots *i* and *j* in the same trial at a specific testing location,


(4) $$K{\left(\cdot |\alpha ,\rho ,\sigma_{e}\right)}_{ij}=\alpha^{2}\exp\;\left(\frac{d_{ij}^{2}}{2\rho^{2}}\right)+\delta_{ij}\sigma_{e}^{2}$$

where *α*, *ρ*, and *σ_e_* are hyperparameters defining the covariance function; *δ_ij_* is the Kronecker delta function with value 1 if *i* = *j* and 0 otherwise; *d_ij_* is the Euclidean distance between centers of the two plots. As this is a Bayesian model, priors for parameters and hyperparameters are required. We adopted weakly informative priors: *t*_3_(0,1) for *α*, *σ* and *σ_e_*; Inv–Gamma(5,5) for *ρ*.

### 2.2 Parameter recovery with NTEP RSM

To ensure that model parameters are identifiable, the following parameter recovery test was performed to evaluate the model. We first generated a synthetic dataset from 3 replications of 10 cultivars rated monthly for 5 years by 5 raters. The entry effects are random draws from a normal distribution with a mean of 0 and a standard deviation of 0.7 (σ = 0.7). Plot location effects are generated from a Gaussian process with an assigned mean vector and covariance matrix with *α* = 0.15, *ρ* = 2.5, *σ_e_* = 0.2. Rating severity is a vector of five evenly spaced numbers over [–0.8,0.8], and category threshold is a vector of eight evenly spaced numbers over [–2,2]. All parameters, functions, and simulated data can be found in the Github repository. The simulated data were fit to the NTEP RSM for parameter recovery.

### 2.3 Linear mixed model

To compare with the existing method, we also implemented the following LMM for each testing location,


(5) Y=η+u+ϵ

in which quality rating, **Y**, was treated as a continuous variable and partitioned into a fixed effect of cultivars, ***η***, and a random effect of rating event, ***u***. ***ϵ*** denotes the residual that the model does not explain.

### 2.4 Model implementation

The NTEP RSM model is implemented in Stan (version 2.29.1) with a Python interface (version 3.10.4). The same model was fitted to data collected from each trial location, and posterior sampling of model parameters was generated by four Markov chain Monte Carlo chains, each with 1,000 iterations. The first 500 iterations were discarded to minimize the effect of initial values, and the rest were thinned by taking every other sample to reduce sample autocorrelation. The convergence of chains was confirmed *via* visual inspection and examining the $\hat{R}$ values of all parameters and the log posteriors. Model codes and output files can be found at https://github.com/QhenryQ/ntep-rsm. The LMM is implemented with the Python package Statsmodels (Seabold and Perktold, 2010).

## 3 Results and discussions

### 3.1 Preliminary data analysis

**Figure 2:**
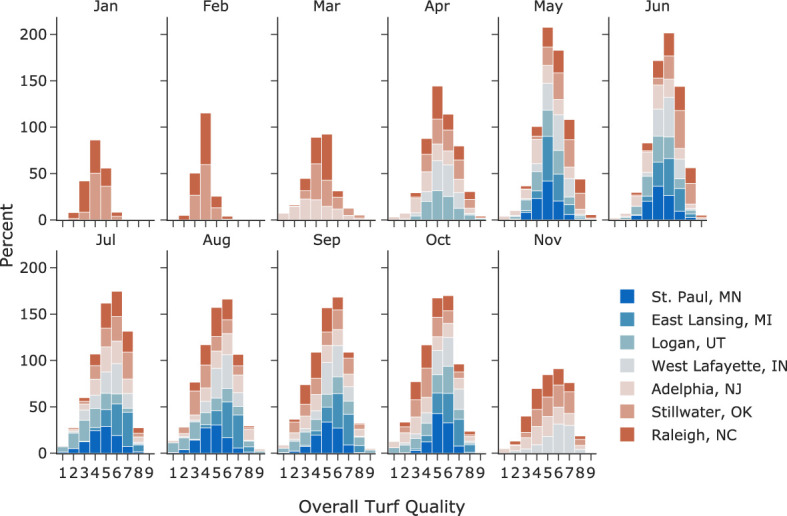
Histogram of raw overall turf quality ratings for each month at seven test locations.

Kentucky bluegrass is a cool-season turfgrass that grows best when temperatures are between 60-75°F and goes dormant in hot, dry summer and cold winter. Given this behavior, turf quality data is only collected from May to October in northern trial locations, while in the southern trial locations, data is usually collected all year round. **Figure 2** presents monthly histograms for all the raw turf quality rating data. In most months, the quality rating showed good symmetry and central tendency around 5 or 6. In January and February, turf quality ratings were only available from Raleigh, NC, and Stillwater, OK. We noticed decreased turf quality ratings and the number of categories assigned in both locations. For example, the February overall turf quality ratings at Stillwater, OK, were found to have a range of [3, 6], with a median of 4. This is presumably due to raters’ adjustment to the dormancy of Kentucky bluegrass. The significant reduction of turf quality in dormancy makes it difficult for raters to distinguish cultivars. Ceiling and flooring effects were also observed at other locations, e.g., the overall turf quality data at East Lansing, MI, and Raleigh, NC, ranged from 2 to 9, while that for data at West Lafayette, IN, from 2 to 8.

### 3.2 NTEP RSM results

#### 3.2.1 Category thresholds

**Figure 3:**
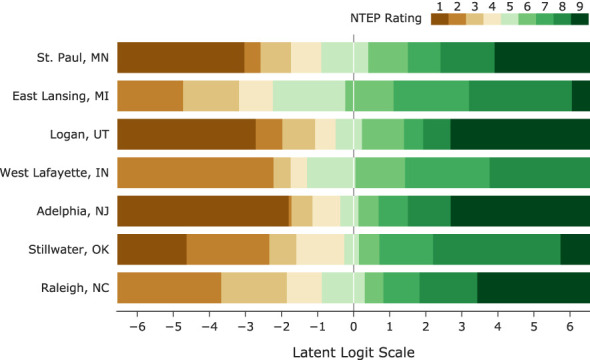
The latent scale partitioned by category thresholds into NTEP rating categories at seven test locations.

“How is Rater A’s 5 different from Rater B’s 5?” This type of question is inevitable when it comes to the comparison of cultivars following the current NTEP procedure. However, such a question cannot be answered without proper definitions of categories, which in our model, are done by identifying category thresholds. These thresholds are points on the latent scale at which a rater is equally likely to select two adjacent response options (Andrich and Luo, 2003). We also assumed there are fixed distances among the category thresholds for raters within the same research group at the same location. This assumption is reasonable given that experienced raters of the same research group usually train newer raters. Estimation of category thresholds from the data provides important feedback on category definitions and how the scale is utilized by each research group, allowing us to ensure raters are adequately differentiating cultivars. When adjacent thresholds are too far apart, a category becomes too wide and less informative; on the other hand, when adjacent thresholds are close, a category becomes too narrow, indicating underutilization of the scale (see Guidelines for Rating Scales and Andrich Thresholds). We examined the non-terminal categories used at seven testing locations (**Figure 3**) . Their widths spanned the range of [0.07, 4.76] on the logit scale, e.g., Category 2 at Adelphia, NJ, only spanned 0.59 logits, while category 8 at Stillwater, OK, was 3.54 logits. Category thresholds are generally required to be in ascending order concordant with the category numbers, i.e., ordered thresholds (Andrich, 2011). Disordered thresholds imply a higher rating may not be assigned as a turf cultivar advances along the scale. Such inconsistency of raters is usually the result of too many options or/and poor category definitions in scale development. Estimated category thresholds from all testing locations, ranging from -6.64 to 6.05, were in order. Large variations were observed in the range of category thresholds. Category thresholds at East Lansing, MI, and Stillwater, OK, spread more than 10 logits, while those in Adelphia, NJ, only spanned 4.5 logits.

#### 3.2.2 Rating severity

**Figure 4:**
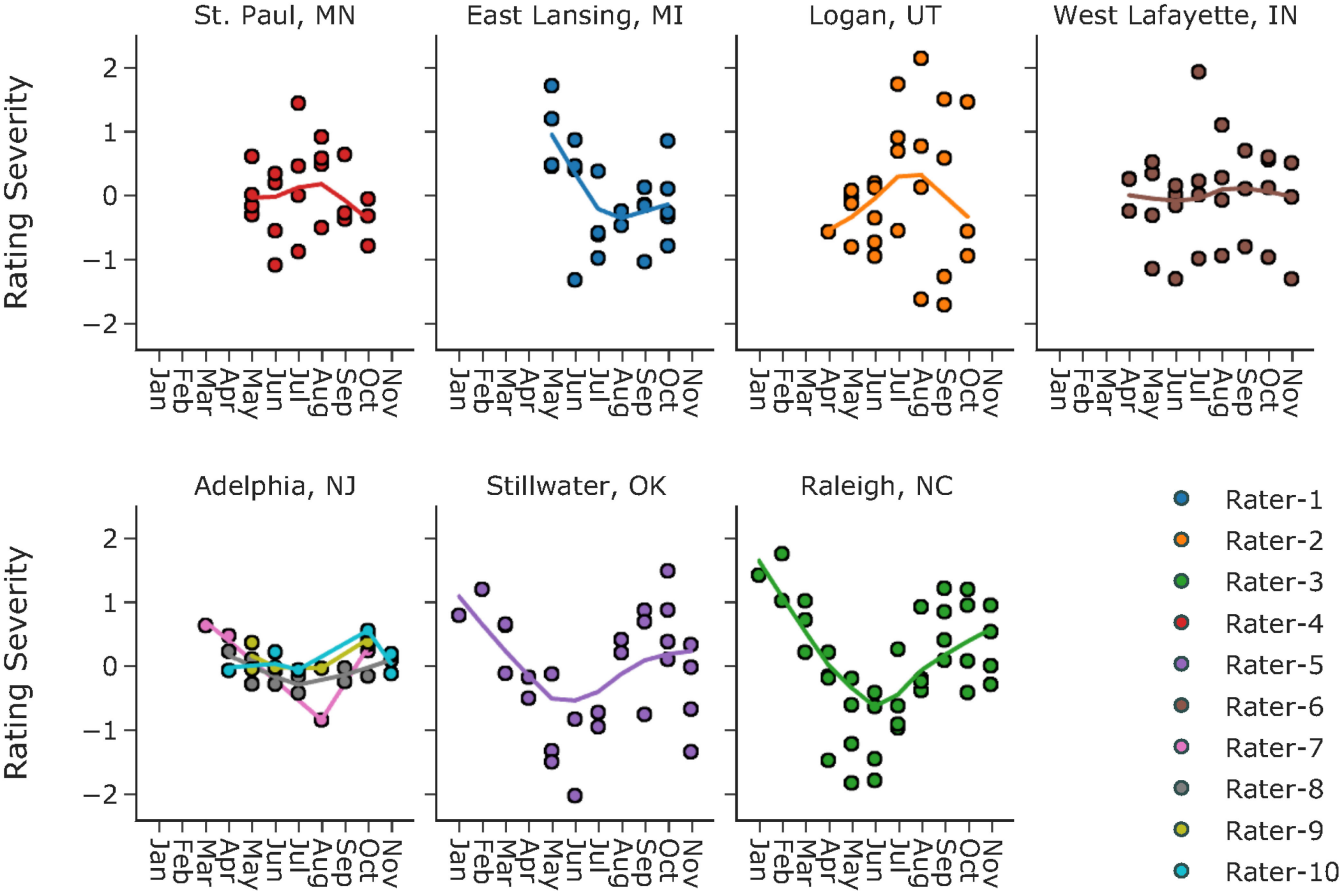
Rating severity estimates and monthly trend lines of ten raters at seven test locations.

Defining category thresholds is not sufficient to answer the question of rater variation. On the constructed latent scale, category thresholds can still slide left (indicating a lenient rating event) or right (indicating a severe rating event). In many fields, severity can be treated as a constant for a given rater. That is to say, whenever the rater conducts a rating, he/she is always the same in terms of severity. However, this might not be true during the evaluation of turfgrass. For new raters, it takes time to achieve consistency; for trained raters, some may adjust their severity to credit cultivars that perform well under harsh environmental conditions or at different times of the year (personal communications with NTEP raters). Historically, there have been two sets of rating criteria for reference standards in NTEP. One is based on an optimal growth environment (e.g., light, temperature, soil moisture) and management regime (e.g., mowing height, fertilization rate), while the other is based on the actual environment or management regime. Using either criterion, the rater must idealize his/her reference standards to compare against all treatments and assign a quality score using a scale of 1 to 9. With the first criterion, we expect consistency of raters regardless of the rating time of the year since the best plot is defined considering all possible growth environments and management regimes. With the second, raters could be either severe or lenient depending on the environment or management regimes at the rating time. We examined the consistency in rating severity estimates of 10 raters who have performed more than 3 ratings across different months. For each rater, we fit a trend line for their rating severity across different months of the year using the weighted scatterplot smoothing (LOWESS) method. No strong trends were observed for raters in St. Paul, MN, West Lafayette, IN, and Adelphia, NJ, while strong seasonal patterns were seen for raters in the other four locations (**Figure 4**). One potential confounding factor in the current definition of rating severity is the seasonality of turfgrass quality. It is also worth noting that while the model focuses on point estimates for the average turf quality, the actual turf quality of cool-season turfgrass is not a constant; instead, it varies over time with strong annual seasonality. Unfortunately, the current data do not provide sufficient information, e.g., the exact rating dates, for investigation on how rating severity changes in response to the seasonality of turf quality. Standard deviations of rating severity per rater ranged from 0.13 to 0.97 on the logit scale. Considering the category widths, such variation in severity for a given rater could lead to changes in rating categories.

#### 3.2.3 Field spatial variation

**Figure 5:**
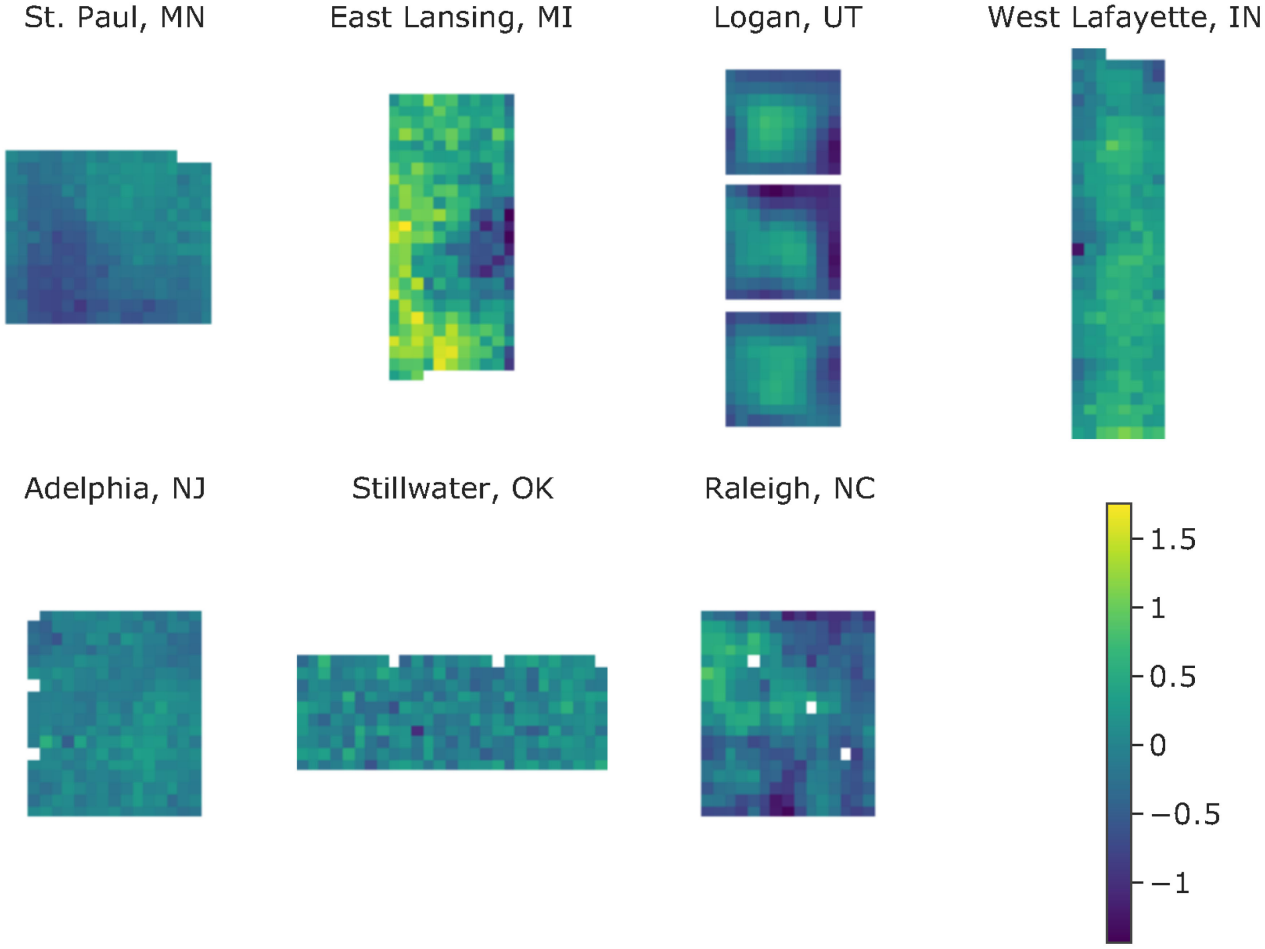
Field spatial variation at seven test locations.

We implemented a Gaussian process to estimate the spatial variation within a specific trial. The traditional cultivar comparison method based on ANOVA or LLM assumes uniform growth conditions within a trial, which is hardly achievable due to heterogeneity in soil texture, seeding depth, elevation gradient, etc. Thus, removing field spatial effect is important for reliable cultivar comparison results. **Figure 5** visualizes the spatial variation estimated by our model at seven testing locations, in which every pixel represents a plot as defined by row and column number. The level of spatial heterogeneity varied from trial to trial; some were higher, e.g., the trial at East Lansing, MI, while some were lower, e.g., the trial at Adelphia, NJ. Noticeably, we observed large edge effects from the trial at Logan, UT, the diagonal division from the trial at St. Paul, MN, and the localized hot spots from trials at East Lansing, MI, and Raleigh, NC. The estimated field spatial variation provided turfgrass researchers with a high-level summary of their trials, which can help improve experimental design and allow better differentiation of cultivars.

#### 3.2.4 Cultivars comparison across testing locations

**Figure 6:**
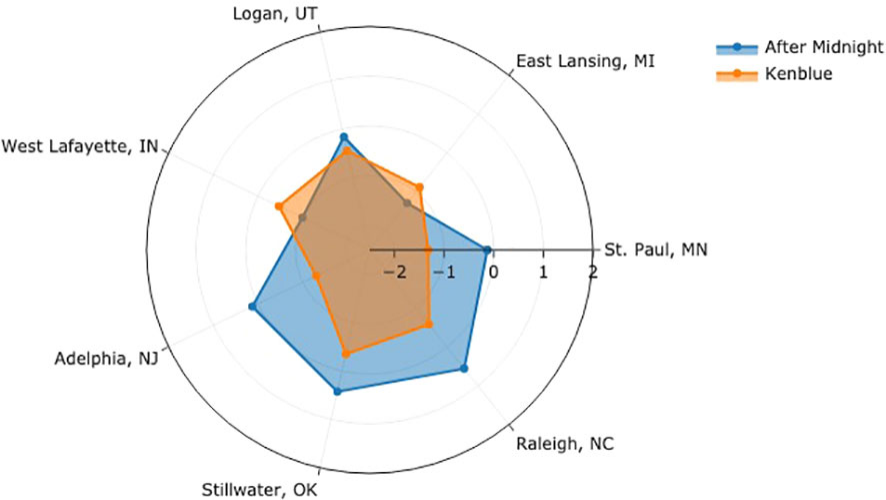
Performance of After Midnight and Kenblue at seven test locations.

Our model quantifies and removes confounding factors at each location, i.e., rating severity and field spatial effect, allowing a more reliable and accurate cultivar comparison. An additional assumption is required for scale alignments to compare cultivars across different testing locations. We assume the average levels for a turfgrass cultivar, as perceived by raters at different NTEP testing locations, are roughly the same. In **Figure 6**, we compared the performance of two example cultivars by aligning the average levels at seven testing locations. Each angular axis represents the latent logit scale at corresponding testing locations, where zero indicates the average level. For ‘After Midnight,’ it performed above average at Adelphia, NJ, Stillwater, OK, and Raleigh, NC, and below average at St. Paul, MN, East Lansing, MI, Logan, UT, and West Lafayette, IN. ‘Kenblue’ performed below average at all locations. When comparing the two, the distance between the logit values estimates how much one cultivar is better than the other at each location. After Midnight outperformed Kenblue at all testing locations except East Lansing, MI, and West Lafayette, IN. The comparison of all evaluated cultivars can be found in **Supplementary Materials** and the GitHub repository.

#### 3.2.5 Effect sizes

**Figure 7:**
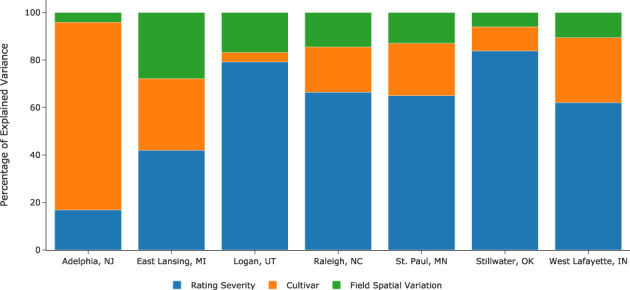
Percentage of explained variance by different effects estimated by the model.

Effect size quantifies the strengths of relationships between variables and determines their practical importance in the study. One way to determine the effect size is by examining the percentage of variance the effects explain. **Figure 7** illustrates the variance percentage explained by the model’s estimated parameters. At all locations except Logan, UT, the effect of field spatial variation is the smallest of the three. In contrast, the effect of rating severity is the largest at all locations but at Adelphia, NJ. Notably, there are seven raters at Adelphia, NJ, compared with 1 to 3 raters at other locations, highlighting the importance of gathering opinions from more raters during cultivar evaluation. The percentage of variance explained by cultivar effect varied drastically, from a merely 4% at Logan, UT, to as much as 79% at Adelphia, NJ. Quantifying and removing these confounding factors is thus essential when evaluating and comparing cultivars in field trials.

### 3.3 Comparison with LMM

The advantages of NTEP RSM over the currently-adopted LMM are three-folded. First, it allows the estimation of additional parameters, namely category thresholds, rating severity, and field spatial variation. All three parameters are essential for rater training, better utilization of the whole scale, and understanding of the field conditions. Second, NTEP RSM separates mean estimations of the evaluated cultivars better. To name a few of the numerous examples, Blue Gem (NAI-13-9), MVS-130, Heartland (NAI-14-187), AKB3241, and RAD 553 all received the same mean estimation of -0.261 at East Lansing, MI, from LLM, while the mean estimates from NTEP RSM were 0.030, -0.020, -0.145, -0.268, -0.580 respectively. Similar patterns were observed for DLFPS-340/3556, Paloma (PST-K13-139), DLFPS-340/3552, J-1138 at St. Paul, MN; DLFPS-340/3556, A16-2, NuRush (J-3510) at West Lafayette, IN; and DLFPS-340/3548, A16-17, Barvette HGT^®^, NK-1 at Logan, UT. Detailed comparison for all cultivars can be found in Among the seven test locations, the largest discrepancies between the two models’ output were seen at Logan, UT. At the same time, the smallest were observed at Stillwater, OK (**Table 2**). It is important to highlight the robustness of the current LMM approach despite all the merits of NTEP RSM. Last but not least, RSM provides more realistic standard deviation estimations, while the currently-adopted LMM generates the same standard deviations for all cultivars at each location. Given the different genetic backgrounds of cultivars, they are unlikely to have the same standard deviations.

**Table 2:** Correlation coefficients between cultivar mean estimates from LMM and NTEP RSM.

Location	Correlation coefficient between LMM and NTEP RSM	
	Pearson’s	Spearman’s rank
St. Paul, MN	0.973614	0.970781
East Lansing, MI	0.928411	0.929173
Logan, UT	0.800883	0.756775
West Lafayette, IN	0.969092	0.955572
Adelphia, NJ	0.997716	0.997600
Stillwater, OK	0.999583	0.999022
Raleigh, NC	0.944150	0.951401

### 3.4 Parameter recovery with NTEP RSM

**Figure 8:**
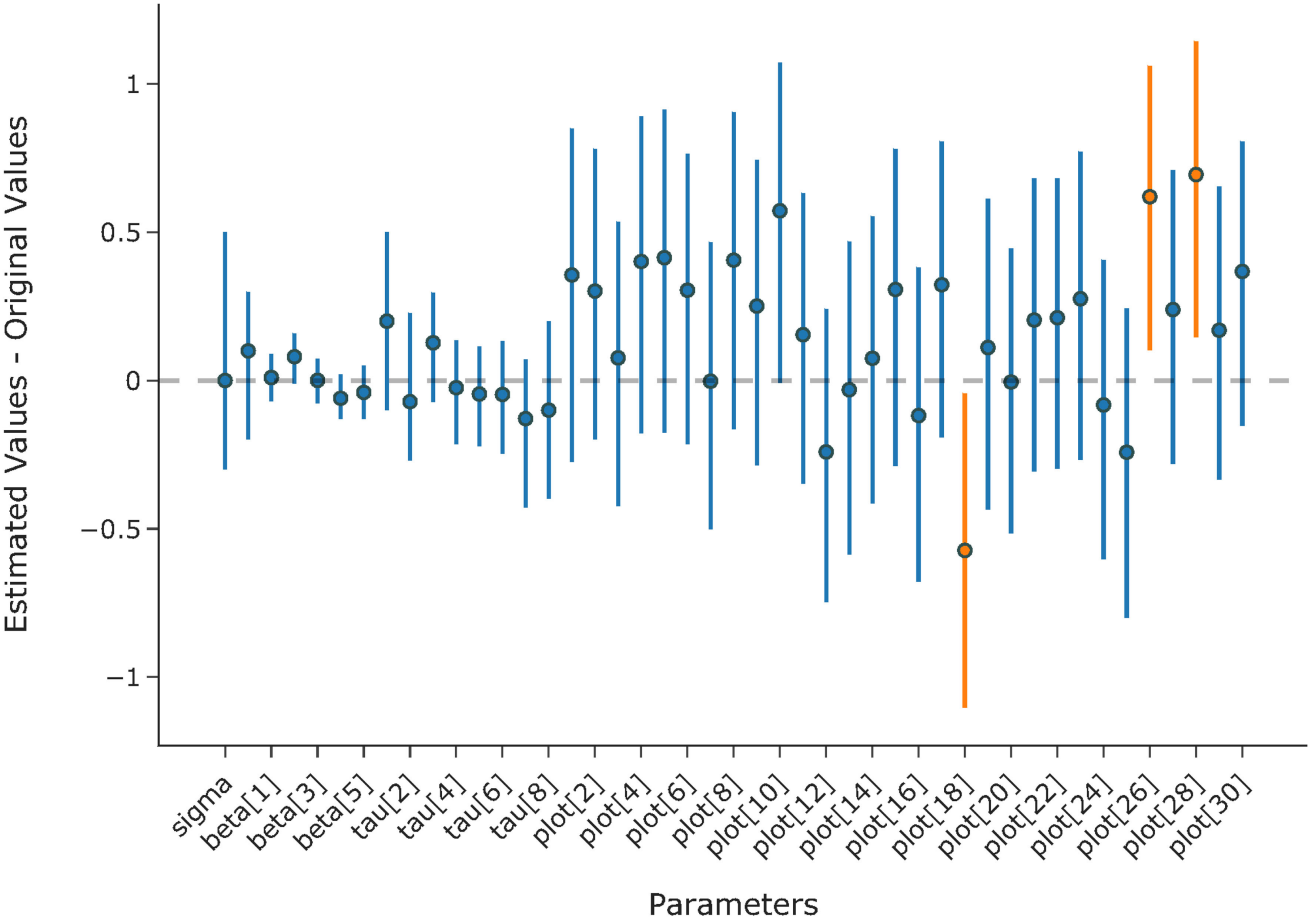
Mean estimation and 95% credit interval for the difference between estimated values and original values of the parameters.

The highest value for $\hat{R}$ was 1.0 for all parameters and the log posterior, suggesting that all four chains have converged. As shown in **Figure 8**, all except three of the 95% credit intervals include zero, indicating the model’s ability to recover the original values of the parameters.

### 3.5 Discussions

Despite the promising results, there are at least two major challenges that lie ahead for the successful implementation of the proposed model. The first and foremost is the lack of data. While NTEP has done a remarkable job of gathering, cleaning, organizing, and storing historical data on cultivar evaluation, a significant amount of valuable data are left out in this process. This includes but is not limited to rater identification, trial layout, rating dates, field gradient, etc. Luckily, researchers generally record and preserve such information at each trial location. Additional work is required to incorporate such data into the current NTEP database. Second, there are too few raters at some trial locations. The fundamental debiasing mechanism of the proposed model is to aggregate individuals’ opinions on the same cultivar into an objective and collective opinion. Multiple raters are required to ensure accurate estimations of the collective opinion on the tested cultivar. As mentioned above, one limitation of the proposed model is the absence of a seasonality component. As a cool-season turfgrass, Kentucky bluegrass thrives during the fall and early spring and slows significantly in growth during the hot summer months. The proposed model focuses on estimating the overall quality for a given cultivar over the entire testing period but cannot provide a quality estimation at a given time of the year. We tested year and month effects as independent Gaussian variables; however, as pointed out by one reviewer, it was unrealistic that months have the same effect across different years. We agree with the reviewer and are exploring better ways to improve the proposed model. A potential approach is the multiple-output Gaussian process model(Li et al., 2021) that incorporates the seasonal grown pattern of Kentucky bluegrass as a prior distribution. This requires additional information on the rating dates. Once implemented, it will allow the analysis of the temporal variation of cultivars, which caters to needs such as mixing/blending cultivars based on spring green up, comparison of cultivars on growth potential at a given time of the year (Woods, 2013). Now that the model assumes raters are consistent in all rating event, we encourage small trial sizes at each testing location. Smaller trials reduce the risk of rater fatigue during rating, thus helping raters to maintain better consistency. For trials with too many cultivars, we recommend ratings be conducted on each replication on separate occasions instead of finishing all the plots at once. Regarding the rating scale, researchers should attempt to achieve a uniform distribution (Bond and Fox, 2013) of category thresholds. NTEP is currently working towards a data ingestion, analysis, and visualization pipeline, with the objectives to provide timely feedback to raters during the reason, to help raters to utilize the rating scale better, and to service a larger audience. NTEP also need to set standards for cultivar average, representing the zero point on the scale, such that results of cultivar comparisons across time and location are accurate and reliable.

## Data availability statement

The datasets presented in this study can be found in online repositories. The names of the repository/repositories and accession number(s) can be found below: https://github.com/QhenryQ/ntep-rsm/tree/main/model_data.

## Author contributions

YQ conceived the idea, developed the model, performed the analysis, and took the lead in writing the manuscript. All authors contributed to the article and approved the submitted version.
