# Supplementary figures and images for: A latent scale model to minimize subjectivity in the analysis of visual rating data for the National Turfgrass Evaluation Program
Source: Front Plant Sci. 2023 Jul 6;14:1135918. doi: 10.3389/fpls.2023.1135918 (PMC10390229; doi:10.3389/fpls.2023.1135918)

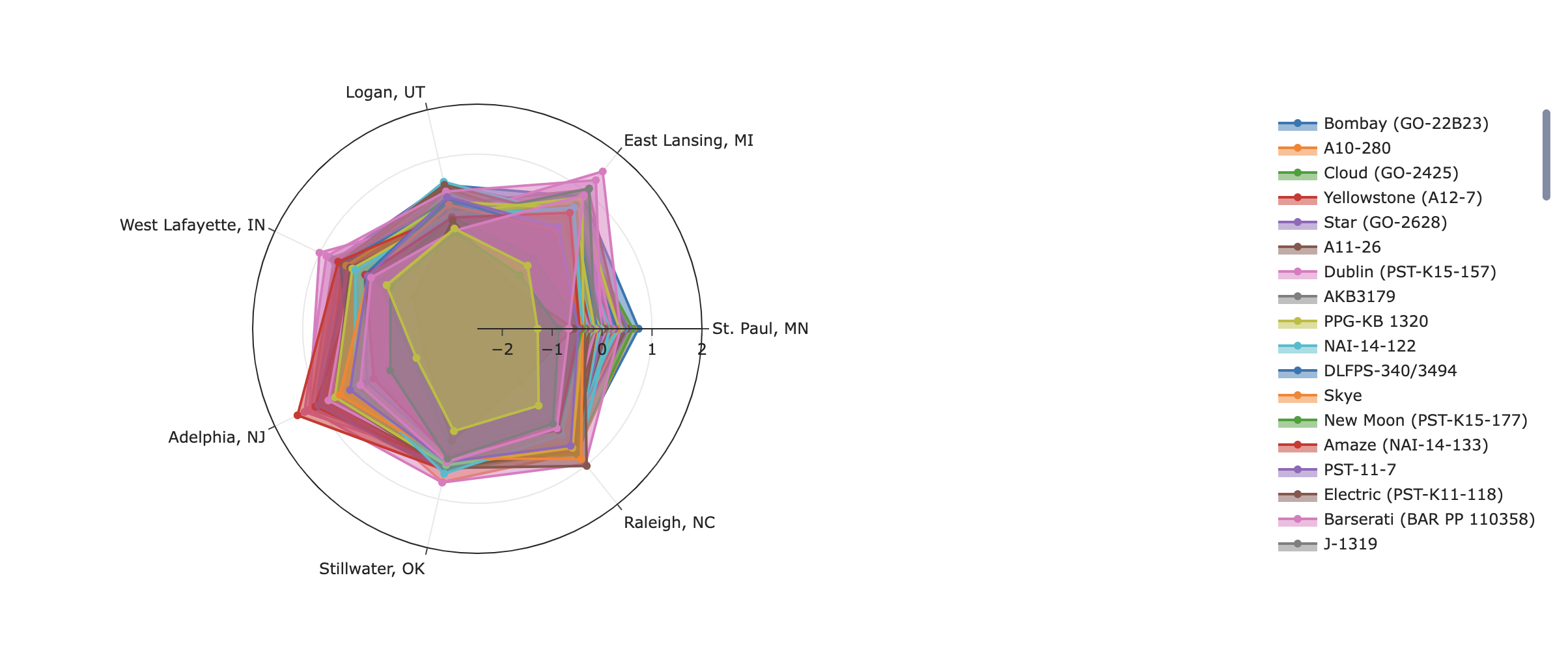

Supplement: Supplementary file 2 [file Image_1.png]
